# Parents of Adolescents Perspectives of Physical Activity, Gaming and Virtual Reality: Qualitative Study

**DOI:** 10.2196/14920

**Published:** 2020-08-25

**Authors:** Lucy McMichael, Nuša Farič, Katie Newby, Henry W W Potts, Adrian Hon, Lee Smith, Andrew Steptoe, Abi Fisher

**Affiliations:** 1 Department of Behavioural Science and Health University College London London United Kingdom; 2 Department of Psychology and Sport Sciences University of Hertfordshire Hertfordshire United Kingdom; 3 Institute of Health Informatics University College London London United Kingdom; 4 Six to Start Redditch United Kingdom; 5 The Cambridge Centre for Sport and Exercise Sciences Anglia Ruskin University Cambridge United Kingdom

**Keywords:** exercise, obesity, video games, adolescent, adolescence, sports, health, leisure activities, virtual reality

## Abstract

**Background:**

Virtual reality (VR) exergaming may be a promising avenue to engage adolescents with physical activity. Since parental support is a consistent determinant of physical activity in adolescents, it is crucial to gather the views of parents of adolescents about this type of intervention.

**Objective:**

This study aimed to interview parents of younger adolescents (13-17 years old) about physical activity, gaming, and VR as part of the larger vEngage study.

**Methods:**

Semistructured interviews were conducted with 18 parents of adolescents. Data were synthesized using framework analysis.

**Results:**

Parents believed that encouraging physical activity in adolescents was important, particularly for mental health. Most parents felt that their children were not active enough. Parents reported their adolescents regularly gamed, with mostly negative perceptions of gaming due to violent content and becoming addicted. Parents discussed an inability to relate to gaming due to “generational differences,” but an exception was exergaming, which they had played with their children in the past (eg, Wii Fit). Specific recommendations for promoting a VR exergaming intervention were provided, but ultimately parents strongly supported harnessing gaming for any positive purpose.

**Conclusions:**

The current study suggests promise for a VR exergaming intervention, but this must be framed in a way that addresses parental concerns, particularly around addiction, violence, and safety, without actively involving their participation. While parents would rather their children performed “real-world” physical activity, they believed the key to engagement was through technology. Overall, there was the perception that harnessing gaming and sedentary screen time for a positive purpose would be strongly supported.

## Introduction

Sufficient physical activity protects against noncommunicable diseases [1] and is associated with better mental health [2]. The detrimental health outcomes associated with low population levels of physical activity have placed a significant strain on the economy and health services [3]. Adolescence, which spans the ages of 10-24 years, encompasses an important phase of social and biological development and has been suggested as the stage at which individuals acquire skills that contribute to future health and wellbeing [4]. Thus, adolescence is an opportune time to encourage engagement with physical activity [5]. Early adolescence is particularly important in this context because physical activity substantially declines from childhood through adolescence and is increasingly displaced by sedentary behavior, particularly screen time [6,7]. Data from the United Kingdom (UK), United States (US), and elsewhere show that less than 20% of adolescents meet the recommended 60 minutes per day of moderate-to-vigorous physical activity (MVPA) [8-11]. Active adolescents are more likely to become active adults and lead healthier lifestyles, gaining benefits both in the short-term (eg, bone health, mental health) and long-term (eg, sedentary behaviors, breast cancer, asthma, and self-esteem) [12,13]. A 2018 prospective cohort of 1826 UK adolescents (followed at 13, 14, and 15 years) found beneficial associations between device-based measures of MVPA and systemic metabolism (metabolic markers such as triglycerides, fatty acids, and systolic and diastolic blood pressure). Associations were more dependent on the most recent engagement, suggesting that regular physical activity sustains beneficial metabolic health and helps prevent disease [14].

### Challenges in Changing Physical Activity in Adolescents

Despite the importance of promoting physical activity in adolescents, it is not yet clear what works. A recent meta-analysis of 17 cluster-randomized trials of school-based interventions, including children and adolescents up to 18 years old, found these did not affect accelerometer-measured MVPA [15]. Multidimensional interventions targeting physical activity across multiple settings, including school/home environments, policy, and parents, could promote, or at least prevent a decline, in physical activity in younger adolescents [15-17]. Despite the potential importance of parental support or involvement in multicomponent interventions [17], few studies have explored parental views, and the possibility of wide-scale implementation of such interventions is unclear. Digital interventions have been proposed as a solution to offer wide-reaching accessibility, but the majority of trials have been small, with mixed findings and web-based [18], which is not necessarily reflective of adolescent digital behavior.

### Gaming Interventions to Change Physical Activity in Adolescents

Gaming constitutes a significant part of an adolescent’s voluntary leisure time behavior, with some estimates of more than 90% playing for at least an hour per day [19]. Gaming is usually sedentary, but games that require bodily movement (ie, “exergames”) have proved popular [20,21]. A 2015 meta-analysis of 35 trials showed that exergames, like Nintendo Wii, increased physical activity and improved physiological parameters as effectively as field-based physical activity and significantly enhanced enjoyment, self-efficacy, and intrinsic motivation for physical activity [22]. Although these small comparative trials were promising, no studies have explored exergaming as a population health intervention, which is likely to involve embedding exergaming in a larger multicomponent intervention that targets social and environmental determinants, including parental support [23-25].

Earlier generations of exergames such as Dance Dance Revolution released in 1998 and Nintendo’s Wii Fit (2007), which sold over 22 million copies worldwide, and more recently, Pokémon Go, downloaded over 800 million times, are examples that have had huge commercial success [20,21]. Research studies on these earlier exergames have shown that playing exergames can increase energy expenditure up to 300% above resting levels and achieve physical activity of at least moderate intensity [22]. Randomized controlled trials in children [25,26], preadolescents [24], adolescents [27-29], and adults [30] found that exergames supported weight loss and increased fitness. However, trials have not been able to confirm exactly which psychological or social factors might lead to long-term engagement [31-33].

### The Potential of Virtual Reality to Increase Physical Activity in Adolescents

Virtual reality has the potential to enhance the impact of exergaming by allowing the user to feel present and immersed in the virtual environment [34]. VR ownership is predicted to rise and estimated to be owned by most homes by 2022 [35]. With the possibility that VR will reach a wider population in the following years, VR hardware and software markets are reported to be increasing to 16 billion US dollars in 2022 from 6.2 billion in 2019 [36].

Small laboratory-based studies in adults have found that VR exergaming increases enjoyment and levels of physical activity but with lower perceived exertion than standard exercise conditions [37-39]. For example, one study involving 88 university staff and students found that although the heart rate was higher in VR than during regular exercise, participants reported feeling less tired and had higher ratings of enjoyment when VR was paired with exercise [40]. These results were echoed in an exploratory pre-post study in which 12 children engaged in VR-based biking (VirZoom) and traditional stationary exercise biking sessions for 20 minutes per session. The results showed no significant differences between the groups in the measures of heart rate. However, perceived exertion during the VR-based exercise was significantly lower, with participants also reporting significantly higher self-efficacy and enjoyment during the VR-based exercise compared to the traditional biking exercise session [38]. Another study of 30 people (between the ages of 6 and 50 years) playing a VR exergame for 15 minutes showed how perceived workout intensity correlated positively with the level of motivation and a significant increase in heart rate after gameplay [39]. These results were similar in a study of 60 females (18-30 years), which tested rowing performance, motivational, and affective impact during an aerobic exercise using VR and non-VR environments. VR groups rated physical activity tasks as more enjoyable, had improved performance (rowing longer distances, particularly the VR group which had a companion avatar) and did not perceive themselves to be exerting more physical effort when they did [37]. Thus, playing VR exergames with companions rather than alone raises and enhances physical activity levels even further. These psychological results, however, should be interpreted with caution due to small sample sizes and a failure to evaluate the effects of long-term physical activity.

Parental involvement is likely an important factor in the uptake of physical activity in younger adolescents [18]. We qualitatively interviewed 31 younger adolescents and determined that they were very interested in the concept of VR exergaming intervention, but that parental approval would be important for implementation [41]. To the best of our knowledge, no studies have explored the wider determinants, such as parental support, that would be required to implement a VR intervention.

The aim of this study, therefore, was to interview parents of adolescents (13-17 year-olds) to understand their views of physical activity, gaming, and head-mounted VR in order to gather evidence and build more understanding around this, in line with the Medical Research Council (MRC) Framework for developing complex interventions [42]. The MRC Framework includes several general stages, including planning, pilot and evaluation stages, reporting, and implementation [42].

## Methods

This study formed part of the development work for the vEngage project [43]. Parents or primary caregivers with an adolescent child aged between 13 and 17 years were eligible to participate. They were recruited through social media, local secondary schools in London (UK), and the University College London (UCL) network and facilities (such as notice boards and online subject pool/study participation announcements) via emails and posters. The aim was to recruit up to 20 participants according to the recommended 10-20 interviews for a medium project using thematic analysis [44]. The study was approved by the UCL ethics committee (Project ID 12669/001), and all participants provided informed written consent before the interview.

### Materials and Procedure

A semistructured interview schedule was developed to guide interviews (Multimedia Appendix 1). According to recommendations from Lewis and Ritchie [45], the schedule was designed to gather rich data on select topics including physical activity, gaming, VR, and the potential for using VR in a physical activity intervention. The schedule included open questions and specific probes to use if required. Interviews were conducted via telephone in June and July 2018 by one researcher (LM). The interviews were recorded and transcribed verbatim before analysis. As part of the interview schedule parents were asked whether their children had any disabilities that might impact their physical activity levels, but this data was not collected using a standardized questionnaire. Data was stored in accordance with the General Data Protection Regulation (GDPR).

#### Analysis

Framework analysis (a form of thematic analysis) was used to synthesize the data. Framework analysis is a systematic and effective approach for analyzing qualitative data in health research [46]. It is a recommended method for data that has been collected in semistructured interviews. Additionally, Framework analysis has been recommended for use when the data relates to a small number of topics and is appropriate for a sample of 10-20 participants [46].

Two researchers (LM and NF) independently analyzed three transcripts and generated a set of codes. The researchers then met to discuss, compare, and adjust the codes in order to develop the final analytical framework. The final framework was used to code all 18 transcripts by one researcher (LM). No particular software was used in coding the transcripts; however, the final set of themes and subthemes were collected with supportive quotes in an Excel spreadsheet (Microsoft). The data were then analyzed to explore emerging themes and identify common themes. Analyses were conducted with guidance from an experienced qualitative researcher (KN). The principal investigator (AF) independently reviewed the Framework Matrix and themes before the final interpretation.

## Results

Participants were recruited through two local secondary schools in South London, UK (n=6), the University College London (UCL) network (n=8), and social media (n=4). Thirteen participants were mothers (age 48-58 years; mean age 53 years, SD 3 years), and five were fathers (age 48-58 years; mean age 52 years, SD 2 years) (see Table 1). Six had sons, two had daughters, and 10 had both. The mean age of all adolescents was 14 years (range 13-17 years, SD 1 years). Interviews lasted an average of 50 minutes (range 30-70 minutes). Only one adolescent had a disability (high-functioning autism spectrum disorder, Asperger syndrome), but this did not impact their ability to perform physical activity as reported by their parent. This difference was important to consider when analyzing our results because the literature on children and adolescents with autism spectrum disorders has shown difficulties in the development of coordination and movement [47]. However, the parent in question reported no issues in their child’s coordination or movement. The final interpretation of the data is summarized in Table 2, with illustrative quotes in the text. Information in parentheses includes the child’s age and gender.

**Table 1:** Demographic characteristics of parents and their adolescent children.

Characteristic	Participants (n=18), n	Parents’ age (years), mean (SD)	Adolescents’ age (years), mean (SD)
Mothers	13	53 (3.4)	14.8 (1.5)
Fathers	5	52 (2.1)	14.6 (1.4)

**Table 2:** Main themes and subthemes.

Main theme	Subthemes
General views on adolescent physical activity	Strong belief in the importance of encouraging physical activity in adolescents
Views on gaming	Adolescents game too often and this can be a dilemma Addiction and violent content were major concerns Accepting technology and gaming as facts of life and embracing potential Peer influence and social pressure are strongest influences
General views on VR^a^	Limited experience VR gaming concerns Concerns would be overcome if health benefits existed
VR exergaming	Concern that a VR fitness game will be a fad Regulations of use
Preference for real-world physical activity	VR exergaming better than nothing Obvious parental support may be off-putting Harnessing screen time for a positive purpose strongly welcomed

^a^VR: virtual reality

### General Views of Adolescent Physical Activity

#### Strong Belief in the Importance of Encouraging Physical Activity in Adolescents

Awareness of physical activity guidelines for adolescents was low. Only one participant knew that the adolescent physical activity guidelines were 60 minutes of MVPA per day. In total, 17/18 (94%) did not know the guidelines, and most were unaware that the guidelines existed. Some participants made accurate guesses.

Parents emphasized the importance of engaging adolescents in physical activity for lifetime health benefits. However, most specifically linked the importance of physical activity to their child’s immediate mental health:

I think it is very [important] because I’m very, very active, and I know how good it is for your mind. (16F)

I think anything that will get her moving more and doing more exercise will be good. (13F)

Only two participants (11%) felt their child was engaged in enough physical activity. Most believed their child “should be doing more” (16F). All strongly believed that it was extremely important to encourage physical activity in adolescents:

Oh, it’s massively important. I would put it one of the highest things, that and eating correctly. (13M)

### Views of Gaming

#### Adolescents Game too Often and This Can Be a Dilemma

All parents (100%) reported their child did some kind of gaming most days of the week, usually reflecting that they felt this was too often and this was associated with a level of guilt: “Probably six out of seven days a week” (16M) and “Every day and far too long. I feel guilty as a parent” (13M).

#### Addiction, Violent Content, and Time Spent Gaming Were Major Concerns

Nearly all participants (17/18, 94%) had negative opinions towards their child’s gaming. All (100%) participants were concerned about gaming being violent. Most participants (14/18, 78%) reported trying to limit gaming, a sentiment generally raised by parents of younger adolescents:

Oh, I absolutely hate it. (14M)

I do think they’re incredibly addictive and then you get issues with trying to manage the time. (13M)

I don’t like the bloody, shooting kinds of things. I think some of it is a little too realistic. (16F)

I wouldn’t be letting him just have it in his bedroom overnight and that kind of thing. (M13)

Participants did perceive some benefits to gaming, including skill development, such as becoming adept at using computers, developing visuospatial skills, and improving cognitive functioning. Another particularly common perceived benefit of gaming was social interaction:

A social aspect to it as well and even though it’s not physical and in-person, there definitely is a lot of banter and a lot of chat, and they’re very much in touch. I find that hugely beneficial. (13M)

#### Accepting Technology and Gaming as Facts of Life and Embracing Potential

Participants felt that due to generational differences, they were unable to relate to gaming. However, 12/18 (67%) participants acknowledged that, when played in moderation, gaming could be fun:

I think it’s just I find it hard to relate to because it’s not something that I do. (13M)

I think in moderation, it is a source of enjoyment. (13M)

Unlike sedentary games, participants shared experiences of taking part in exergames with their children. Only 2/18 (11%) participants reported positive experiences with exergames such as the Wii Fit (Nintendo, 2006), but these were referred to as something done in the past. Despite concerns around gaming, participants accepted that gaming and technology were parts of life and could be used as a force for good. Participants felt that they could not fight against technology, that it was a reality of modern life:

It feels like an ancient device now, but at the time, it was one of those things that came along, and everybody was really excited about it. (13M)

Technology is here, it’s not going away, and that’s fine if it has benefits for his health, then, yes, I’d always be up for that. (14M)

#### Peer Influence and Social Pressure Are Perceived as the Strongest Influences in Adolescence

A majority of participants (10/18, 55%) highlighted the social pressure on adolescent gaming, particularly in terms of owning certain consoles and games. There was a general view that adolescents were influenced by peer and social pressure, then persuaded parents, rather than the reverse. Nearly all participants (17/18, 95%) underlined the role that the peer group plays in adolescent interests:

So that’s the thing is to try and get something that becomes the really cool thing to do. (14M)

### General Views of Virtual Reality

#### Limited Experience

Most participants (15/18, 83%) had only a limited understanding of VR, and most had not tried it. A few had tried phone-based headsets. The same number of participants expressed an interest in VR, and some said that it seemed exciting. Others recognized it had potential:

It seems like you’re actually in there, and you’re moving within that space. And it’s a whole lot more realistic, you’re actually in the game rather than watching. (13M)

I think it opens possibilities, definitely that’s something I think that’s really the future. (17F)

#### Virtual Reality Gaming Concerns

A majority of participants (13/18, 72%) were more concerned about violent games in VR than in regular gaming:

Involving killing people, and that would not be appropriate in a virtual reality kind of scenario. (13M)

It’s quite a weird idea that you then have no idea what it is they’re experiencing. (13M)

Similarly, participants expressed safety concerns around multiplayer VR games as they may be unaware of whom their child is interacting with:

That she might be playing or getting in contact with people who she doesn’t actually know. (13F)

Further health and safety issues that participants mentioned concerned the practical matter of the space that VR would require.

I wouldn’t want it taking over the sitting room. (16M)

While participants suggested that gaming could be a social activity, they were concerned that VR gaming could be isolating:

It seems solitary, another solitary thing that, she’ll be up in her room on her own. (13F)

All parents cited cost as a barrier:

Obviously, there’s the cost of it. (13M)

#### Concerns Would Be Overcome if Health Benefits Existed

A third of the participants (6/18, 33%) suggested they would overcome their concerns if VR presented a tangible health benefit. Participants recognized that outside of gaming, VR could have the potential to be educational, and this was viewed as a positive thing:

If they (parents) can see the benefits of technology, then they’re quite happy to invest in it. (13M)

I’m sure there’ll be some educational aspects to it that it could be used for as well. (13M)

### Virtual Reality Exergaming

#### Concern That a Virtual Reality Fitness Game Will Be a Fad

Participants did express concern that a VR fitness game could be a fad or novelty that comes and goes (7/18, 39%). However, participants suggested that if the game could maintain their child’s interest, they would be more likely to invest:

It might be one of those fad things—they use it all the time for the first month, and then it’ll die off slightly. (16F)

Providing that it has some kind of stickiness in terms of it wasn’t just a five-minute thing. (13M)

#### Regulation of Use

A few participants (3/18, 17%) expressed that it would be important to regulate and manage the usage of the intervention. A majority of parents (13/18, 72%) expressed that they used technology and did not want to appear hypocritical:

It would need to be regulated really so that it’s not just taking over. (16M)

I think it’s fine. I mean, we use technology for everything now, don’t we? So it’s an inevitable march. I’ve got no problem with it. I’d be a bit of a hypocrite if I did, considering I work in technology. (F14)

### Preference for Real-World Physical Activity

#### Virtual Reality Exergaming Better Than Nothing

Half of the participants (9/18, 50%) felt strongly that physical activity in the real world would provide greater benefits. Participants showed a preference for outdoor activities and team sports. Nearly all of the participants (17/18, 94%) thought their child would be excited to try a VR fitness game, and that they would embrace it:

I would see it as inferior to physical activity in the real world. (13M)

I feel they’re sitting indoors, on the computer, when they could be outside doing other things. (14M)

They love new things, and they love the next step up technology-wise. He would love it, I’m sure. (13M)

#### Obvious Parental Support May Be Off-Putting

However, 5/18 (28%) participants perceived that adolescents might be less keen to engage in something that their parents were actively encouraging. Participants also expressed the view there may be gender differences relating to a VR fitness game, such that boys may be more engaged than girls and that girls may engage differently.

I think that all teenagers and young people are developing a separation from their parents, and not necessarily wanting to do what their parents say. (13M)

Maybe girls wouldn’t be as into it as boys or maybe the time you have to give, you know, different challenges, different games. (14M)

Participants also had ideas as to what may make the game appealing. These included levels, competition, a social element, and a challenge that requires skill:

I suppose some kind of score, so you could either beat people or beat your own score. (16F)

Participants also reflected on the fact that adolescents expect the best in terms of quality and graphics:

They are tough consumers. If it’s not on-brand, if it’s not hitting their buttons, then they will just drop it and say, or won’t even pick it up. (13M)

#### Harnessing Screen Time for a Positive Purpose Strongly Welcomed

All participants welcomed the potential of gaming with a positive purpose, particularly mentioning being active during screen time:

If somebody has already lost them to screen time, having part of their screen time as exercise could be fantastic couldn’t it? (13M)

## Discussion

### Principal Findings

All participants in our study strongly believed that encouraging physical activity in adolescents was important. Most participants felt their children were not active enough. National datasets in adolescents using objectively measured physical activity suggest this perception is likely correct, with less than 15% meeting minimum guidelines [48]. Awareness of the recommended physical activity levels for adolescents was very low in our sample, mirroring the findings of our qualitative study in 31 adolescents [41] and a quantitative survey by our group in >1000 families showing that less than 20% of parents knew the recommended physical activity guidelines for their preschool children [49]. Parents who were aware of physical activity guidelines for their children were more likely to be supportive of physical activity [49], suggesting parental education ought to be incorporated in an intervention. Knowing targets for health would also assist with goal setting, a behavior change technique consistently associated with successful physical activity change [50].

Parents tended to have negative perceptions of gaming, particularly expressing concerns about addiction. Reflecting and perhaps exacerbating parents’ concerns, “gaming disorder” is recognized in the International Classification of Diseases (ICD-11) [51], highlighting the scale and seriousness of addiction and possibly putting further pressure on parents to be alert to their children’s gaming habits. The participants reported that trying to monitor their children’s gaming and any digital intervention involving gaming would have to address this conflict. Positively using gaming time would be welcomed, so a possibility is that games designed in the future, used as physical activity intervention, could include materials and messages around replacing sedentary screen time with active gaming. A future game could also encourage breaks, and include real-world elements such as trying various sports or visiting clubs (eg, climbing wall, boxing, trampoline park). Parents in our study suggested that in an ideal world, their child would be encouraged to be active outdoors, but acknowledged that the gaming element would engage their child.

Parents reported they managed their child’s gaming to some extent (usually with time limits), but acknowledged that adolescents should be allowed some autonomy, particularly as they approach young adulthood. This impression was supported by a study of 500 families using latent growth curve monitoring to demonstrate that parental media restriction decreases throughout adolescence [52]. Except for exergames, parents felt unable to relate to gaming, attributing this to a generational gap. The same effect was reported in a study of 80 teens aged 16-18 years and their parents exploring the mediation of mobile phone and internet use [53]. Exergaming was an exception in our study in terms of parental involvement. Almost half of the participants had used exergames, such as the Wii Fit in the past. Gamification of physical activity has been proposed as a way to encourage the integration of physical activity into their daily lives [54]. Those parents who tried exergames reported playing exergames with their children, and reported enjoying it (but recognizing that the technology was now outdated and was viewed as “past activity”). Playing with others was an important driver, including peer and social influence, and whether the game was believed to be “cool” [55].

### Parents Had Limited Knowledge About Virtual Reality

Harnessing novel technology, such as the mobile game Pokemon GO, social media interventions and immersive VR games have been identified as an effective way of increasing physical activity in adolescents [54,56,57]. Therefore, the intervention should attempt to alleviate the discomfort that some experience when faced with new technology. For example, an information leaflet for parents enclosed in the intervention may help reduce parental concern.

Parents identified several benefits to gaming. These included socializing, becoming skilled at using technology, moving, or utilizing screen time for health benefits, the development of visuospatial skills and strategy. Participants were aware of VR but had a limited understanding of its use. Perceptions of VR were generally positive, with many participants describing it as interesting, intriguing, and as having potential. However, there was uncertainty around VR and its applications, and participants expressed concern about their child using violent games would be used in VR and have damaging psychological effects. Therefore, when developing the intervention, these factors should be considered, with violence avoided and, for example, providing guidance to help protect against addiction.

Parents reported they managed their child’s gaming to some extent (usually with time limits) but acknowledged that adolescents should be allowed some autonomy, an effort supported by a study of 500 families using latent growth curve monitoring to demonstrate that parental media restriction decreases throughout adolescence [58]. Participants raised health and safety concerns, particularly around multiplayer games, with the belief that they may cause adolescents to interact with people they do not know. There was also concern about using a VR headset, causing injury or isolation for their child. Cost was a barrier reported by all participants. Many were not aware of the cost but believed it to be prohibitively expensive. The concern is not surprising given that VR is not yet mainstream technology, despite the predicted increase in household ownership by 2022 [36]. It is important that in the intervention development concerns are recognized and addressed by ensuring appropriate protective measures are taken, such as education around VR and safety.

### Health Benefits of Using Head-Mounted Virtual Reality for Physical Activity Changed Parents’ Attitude Toward Gaming

Despite concerns such as “novelty wearing off in 5 minutes,” participants said they would support their children’s use of VR if they knew there was a health benefit or educational element. Thus, despite reservations around VR, parents see the potential of the technology and would be open to it should it present a tangible health benefit. While some participants seemed to embrace technology and its potential for promoting health and fitness, others appeared merely accepting of technology and surrendering to its applications. These responses show that, while in different ways, all parents were open to using technology to improve the health of adolescents [58]. With the studies mentioned in the introduction, there is a potential risk of publication bias because studies showing positive or negative results are more likely to be published than those that show no results [18].

Parents reported a strong preference for real-world physical activity and felt that exercising using VR would be “better than nothing.” There was a strong message for VR having the potential for adolescents who do little or no physical activity, which is the majority [59]. Therefore, an effective intervention is required, and a home-based physical activity intervention could particularly appeal to adolescents [6]. The intervention itself could also come with the direction that it is to be used in combination with outdoor exercise, which adolescents have previously suggested would be effective and appealing [41].

It may be important that the game is marketed directly to adolescents rather than to their parents since adolescents strive for independence [56]. Parents also suggested features they thought their children would find appealing in a game. Competition, levels, a challenge, and a social element were the aspects that came up most often and reflected findings from previous gaming research [60,61].

Only one adolescent child had a known disability, Asperger Syndrome. It was interesting to see that the mother of this child stated that she believed that gaming and head-mounted VR would help her child because “it takes away the pressure of having to interact with other people” (13M). Her feedback was interesting, but due to the aims of this study, we did not explore this further. While we recognize the potential for the benefit of exergames in children with disabilities, we felt that any disability that impacted movement might impact parent’s perceptions of physical activity. Exergaming would best be excluded from the scope of our study.

### Strengths and Limitations of the Study

The sample size was sufficient, according to Clarke and Braun’s [41] recommendation of between 10 and 20 participants. It is not possible to say if the theoretical saturation was reached because we cannot be sure that the views of parents would differ in other populations [62,63]. Themes that reached saturation included all major themes, especially lack of experience with VR, general views on physical activity, worries around violent content, spending too much time online or gaming, and preferences for real-world physical activity.

Our sample comprised of more mothers than fathers and more parents of boys than girls, despite there being low levels of physical activity in both adolescent girls and boys. It would be useful to investigate whether parental concerns differ depending on the child’s gender. Further research should also explore ways to engage boys and girls with the intervention equally. Social desirability bias may affect participants’ answers since parenting methods can be a sensitive topic, but participants were open about how they knew their child should be engaged in more physical activity.

### Conclusions

Adolescents have previously raised parental support as being an important factor in the intervention success [41], consistent with findings from a meta-analysis that found parental support correlated with adolescent physical activity [64].

The results of this study provide support for developing a head-mounted VR intervention to promote physical activity in adolescents. The findings provide useful insight for intervention development. Parents had negative associations with gaming but are accepting of it and embracing its potential. It is important that concerns are considered in the intervention development and negated, where possible, to maximize adoption and, ultimately, the efficacy of the intervention. Overall, parents believe encouraging physical activity in adolescents to be of importance. Therefore, while parents have reservations, it seems they would welcome anything that may improve their children’s health, including if it involves harnessing a behavior like gaming and using it as a force for good. Recommendations for the next stage of intervention development would be to further research how best to educate and inform parents to reduce uncertainty around VR and the intervention. Additionally, shaping the game with an adolescent steering committee is recommended in order to ensure it is enjoyable and has longevity.

## Appendix

Multimedia Appendix 1 Interview schedule.

## Abbreviations

ASD: autism spectrum disorder
GDPR: General Data Protection Regulation
MRC: Medical Research Council
MVPA: moderate-to-vigorous physical activity
VR: virtual reality
