# Supplementary material for: Parents of Adolescents Perspectives of Physical Activity, Gaming and Virtual Reality: Qualitative Study
Source: JMIR Serious Games. 2020 Aug 25;8(3):e14920. doi: 10.2196/14920 (PMC7479580; doi:10.2196/14920)
Supplement: Multimedia Appendix 1 [file games_v8i3e14920_app1.docx]

Appendix A – Interview Schedule

Section 1: Informed consent and demographics (10 mins)

Read information sheet, check they understand, respond to any questions, and seek consent

Check eligibility

A parent of a child aged between 13-17 years

Record demographics of participant and their adolescent (on Data Collection Sheet)

Age

Gender

Section 2: Physical activity (10 minutes)

The purpose of this section is to understand parental opinions about the importance of physical activity, specifically relating to the physical activity levels of their child. It is also an opportunity to build rapport.

In a typical week, what types of physical activity does your child engage in, that you are aware of?

How often do they do that?

How intense is that exercise?

What do you believe to be the recommended levels of physical activity for an adolescent?

Do you think your child does enough physical activity?

Section 3: Gaming and virtual reality (25 minutes)

What do you think about gaming, such as on a computer, PlayStation or Xbox?

How often do you think your child games?

How do you feel about your child spending time gaming?

What is your current understanding of ‘Virtual Reality’? [if none give a brief explanation/description and show image]

What do you think about virtual reality/ What’s your current opinion of it?

Probe: Why do you say that /Why do you think this?

How would you feel about your child using virtual reality?

What do you think about using virtual reality in gaming?

What is your opinion of using virtual reality in the home?

What concerns would you have about your child using virtual reality for gaming at home?

Are there any barriers that you can think of that may prevent you purchasing virtual reality equipment for use at home?

What would encourage you to purchase virtual reality equipment for use at home?

Section 4: Linking IVR and PA (25 minutes)

What is your opinion on your child using technology for the purpose of keeping fit and healthy?

Do you have any concerns about your child using virtual reality to keep fit and healthy?

How do you think your child would react towards an intervention or game such as this?

Do you foresee any particular issues with their usage of the intervention?

Would you consider purchasing the VR headset and equipment for a physical activity game?

Have you heard of any technology designed to make the user more active?

Probe: Get them to think a bit outside the box, even if movement isn’t their main aim, e.g. Pokemon Go, Dance Central, Wii Fit, Xbox Kinnect

Outline ideas such as Wii Fit or Dance Central

What do you think about this type of technology?

Probe: how much of a role do they think technology plays in sport?

How important do you think it is to encourage physical activity in adolescents?

How might you do this?

What features do they think would make such a programme appealing to them?

How do you think parents such as yourselves would feel about this kind of game?

Outline problem with contradiction in encouraging screen-time when promoting physical activity – most interventions try to reduce screen-time – could this intervention send mixed messages?

Do you think encouraging young people to use technology to exercise more might be confusing?

Do you have any other thoughts or concerns about using virtual reality to encourage physical activity in adolescents?

Section 5: Debriefing and answer any questions (5 minutes)
